# Diagnosis and management of small bowel obstruction in virgin abdomen: a WSES position paper

**DOI:** 10.1186/s13017-021-00379-8

**Published:** 2021-07-03

**Authors:** Yousef Amara, Ari Leppaniemi, Fausto Catena, Luca Ansaloni, Michael Sugrue, Gustavo P. Fraga, Federico Coccolini, Walter L. Biffl, Andrew B. Peitzman, Yoram Kluger, Massimo Sartelli, Ernest E. Moore, Salomone Di Saverio, Esfo Darwish, Chikako Endo, Harry van Goor, Richard P. ten Broek

**Affiliations:** 1 Department of Surgery, Radboud University Medical Center, P.O. Box 9101, 6500 HB Nijmegen, The Netherlands; 2 Department of General Surgery, The Baruch Padeh Medical Centre, Poriya, Israel; 3 Second Department of Surgery, Meilahti Hospital, Helsinki, Finland; 4 Department of General Surgery, Parma University Hospital, Parma, Italy; 5 General Emergency And Trauma Surgery, Bufalini Hospital, Cesena, Italy; 6 General Surgery Department, Letterkenny Hospital, Letterkenny, Ireland; 7 Faculdade de Ciências Médicas (FCM), Unicamp Campinas, São Paulo, Brazil; 8 Acute Care Surgery, The Queen’s Medical Center, Honolulu, Hawaii USA; 9 Department of Surgery, Trauma and Surgical Services, University of Pittsburgh School of Medicine, Pittsburgh, USA; 10 Division of General Surgery, Rambam Health Care Campus Haifa, Haifa, Israel; 11 Department of Surgery, Macerata Hospital, Macerata, Italy; 12 Trauma Surgery, Denver Health, Denver, CO USA; 13 Addenbrooke’s Hospital, Cambridge, UK

**Keywords:** Small bowel obstruction, Virgin abdomen, Adhesions, Conservative management, operative management, Immediate intervention

## Abstract

**Background:**

Small bowel obstruction (SBO) is a common surgical emergency, causing high morbidity and healthcare costs. The majority of SBOs are caused by adhesions that result from previous surgeries. Bowel obstruction, however, also occurs in patients without previous operation or known pathology, a so called virgin abdomen. It is unknown if small bowel obstruction in the virgin abdomen (SBO-VA) can be managed according to the same principles as other cases of small bowel obstruction. The aim of this position paper is to evaluate the available evidence on etiology and management of small bowel obstruction in the virgin abdomen.

**Methods:**

This is a narrative review with scoping aspects. Clinical topics covered in this review include epidemiology and etiology of SBO-VA, diagnosis and imaging, initial assessment, the role of surgical management in SBO-VA, and the role of non-operative management in SBO-VA.

**Results:**

Our scoping search revealed seven original studies reporting original patient data related to SBO-VA. All the included studies are retrospective cohorts, with populations ranging between 44 and 103 patients with SBO-VA. Adhesions were found to be the cause of the obstruction in approximately half of the reported cases of SBO-VA. A relatively high number of cases of SBO-VA were managed surgically with studies reporting 39–83%. However, in cases where a trial of non-operative management was started, this was generally successful.

**Conclusion:**

The data available suggest that etiology and treatment results for patients with SBO-VA are largely comparable to the results in patients with SBO after previous abdominal surgery. We therefore propose that patients with a virgin abdomen could be treated according to existing guidelines for SBO and adhesive small bowel obstruction.

## Background

Small bowel obstruction (SBO) is a common surgical emergency, accounting for almost 50% of all emergency laparotomies with significant in-hospital morbidity and costs [1–4]. Most patients with SBO have undergone previous abdominal operations (80%) [5, 6], with adhesions as the single most common cause (60–75%) of SBO [1, 7]. However, SBO also occurs in patients who had no prior abdominal surgery, referred to as a virgin abdomen.

In the past decade, a paradigm shift in the treatment of SBO in patients with previous abdominal surgery has been implemented. Today, the majority of small bowel obstructions are managed non-operatively, treatment comprising bowel decompression, water-soluble contrast agents, and fluid resuscitation. Non-operative management has been found safe and efficacious in 70% of SBOs caused by adhesions (ASBO) [8]. Many authors, however, suggest that surgical exploration is still mandatory in the case of SBO in the virgin abdomen (SBO-VA), based on the assumption that SBO-VA is usually caused by other etiologies than adhesions, such as malignancy, internal hernia, and bezoars as the most prominent causes [9, 10].

On the other hand, recent studies suggest a high incidence of adhesions also in patients with SBO-VA [6, 11]. The origin of adhesions in the virgin abdomen can be congenital, or the results of (unrecognized) abdominal inflammation in the patients’ history [12]. The observation that most SBO-VA are caused by adhesions could have important implications for treatment, signifying that guidelines on the management of ASBO might also apply to the majority of patients with SBO-VA.

The present paper aims to provide an overview of the literature on the etiology and management of SBO-VA and to generate recommendations for clinical management and future research.

## Methods

This is a narrative review with scoping aspects [13]. A formal systematic review and meta-analysis were not feasible due to the many aspects of diagnosis and management of SBO-VA we would like to cover. Moreover, because of the paucity of literature, we will also discuss papers that might not meet strict inclusion criteria as applied in a systematic review.

Therefore, a scooping review search framework was found more appropriate to guide our position paper. In contrast to systematic reviews, where a focused research question with narrow parameter should be chosen, a scoping search allows for a broad research question, adjusting the inclusion and exclusion criteria according to available literature, and the quality of the chosen studies is not a priority. Data might be extracted mainly for qualitative assessment; the quality of the studies found and the data extracted are used to identify gaps in the literature.

Our scooping study followed the 5-step pathway as described by Arksey and O'Malley framework [14]:

1. Main questions
  What is the etiology of SBO-VA?
  What are the outcomes of operative management of SBO-VA?
  Is a non-operative trial appropriate in patients with SBO-VA?

2. Search strategy for relevant topics

PubMed, the Cochrane database, EMBASE database, and Google Scholar were searched to March 2020 without date restrictions. PubMed studies were identified using the following MeSH terms: “intestinal obstruction,” “small intestine,” “ileum,” “jejunum,” and “aged” and keywords “small bowel obstruction,” “Virgin abdomen,” “non-operative management,” “water-soluble contrast agents,” and “Gastrografin.” Similar search strategies were performed in Embase and Google scholar. The Cochrane database was searched for all articles relating to small bowel obstruction. After performing the different searches, duplicates were identified and deleted.

To increase the yield of our search to detect relevant publications, we also searched reference list of relevant articles and guidelines for the treatment of patients with SBO.

3. Relevant studies selection

All the studies that included data for a (sub)cohort of SBO-VA patients relating to our search questions were included.

4. Data extraction

From included studies, we extracted data related to clinical topics including epidemiology and etiology of SBO-VA, diagnosis and imaging, initial assessment, the role of surgical management in SBO-VA, and the role of non-operative management in SBO-VA. All the relevant data was charted in tables.

5. Collation, summarizing, and reporting

The charted data was combined, categorized, and compared across the included studies.

All relevant information was reported and discussed to answer the study questions.

### Definitions

#### Small bowel obstruction

Small bowel obstruction is a surgical emergency in which a mechanical obstruction of the small intestine hinders the passage of intestinal contents. Typical symptoms of small bowel obstruction are abdominal pain, vomiting, distension, and constipation [8]. However, not all these symptoms may be present especially in the elderly population [15].

Small bowel obstruction can be classified as partial (incomplete) or complete obstruction. Imaging studies such as water-soluble contrast agents (WSCA) or CT can help differentiate between complete and incomplete obstruction. In case of a partial obstruction, there is a higher likelihood that a bowel obstruction can be managed by non-operative means [16, 17].

#### Virgin abdomen

The term “virgin abdomen” refers to the abdomen of a patient without prior surgery, radiotherapy, or known peritoneal inflammatory disease in history. There is no consensus in the literature about primary abdominal wall hernias that are not related to a previous incision [6, 18]. In our results, we will describe whether patients with a primary abdominal wall hernia have or have not been included among patients with virgin abdomen.

#### Peritoneal adhesions

Peritoneal adhesions are defined as fibrous scar tissue that connects surfaces of intra-peritoneal organs that are normally separated.

Peritoneal adhesions may be classified as congenital or acquired [19]. Most adhesions are acquired and result from the peritoneal healing response upon injury, typically abdominal operation [20]; other less common etiologies of acquired adhesions include malignancy, radiotherapy, abdominal or pelvic inflammations, and endometriosis [8, 12, 21].

Congenital adhesions are defined as anomalous intra-peritoneal adhesions that are not related to a previous abdominal disease or operation; these might result as remnants of physiological organogenesis [22, 23].

## Results

Our scooping search revealed seven original studies reporting original patient data related to SBO-VA. All the included studies are retrospective cohorts, with populations ranging between 44 and 103 patients with SBO-VA. Six studies are single-center, and one study was a multicenter cohort.

Five studies reported data on the etiology and operative findings in patients with SBO-VA [6, 11, 18, 24, 25]. One of the studies additionally compared the operative findings between patients with SBO-VA and patients with SBO after previous abdominal surgery [25].

The accuracy of computed tomography in identifying the cause of the SBO was discussed in 2 studies, in which the findings in computed tomography were compared to the operative findings [6, 11].

Data on the treatment options were discussed in 6 studies, including a comparison between operative management and non-operative management [6, 11, 18, 24, 26, 27]. In 2 studies, water-soluble contrast agents (WSCA) were used in the non-operative management [24, 27].

Meanwhile data on recurrence, follow-up, and further investigations were reported in 4 studies [6, 11, 18, 26].

### Epidemiology and etiology

In three cohort studies, SBO-VA accounted for 5–16% of all cases of SBO [6, 24, 27]. Most of these cohorts were single center, and the number of patients with SBO screened was roughly between 600 and 850 in each studies. Population-based studies are lacking.

Based on the available data, SBO-VA seems more common among males (65–83%) with a median age of diagnosis between 58 and 65 [6, 26]. Between 39 and 83% of the patients with SBO-VA were reportedly treated by operation [6, 11, 18, 26], which compares high to the number of operations performed in recent cohorts of SBO patients who did have prior surgery [8].

Several recent studies retrospectively analyzed the etiology of small bowel obstruction in patients with SBO-VA. Among patients operated for SBO-VA, etiology of SBO was adhesions in 26–100% (Table 1) [6, 11, 18, 25–27]. In two studies, 75–100% of patients conservatively treated were diagnosed with adhesion [6, 11]. In total, adhesions were the cause of bowel obstruction in 134/280 (47.9%) cases of SBO-VA reported in the studies included (Table 1).

**Table 1:** Etiological findings in 6 studies on ASBO-VA

	Beardsley et al., 2014 [6]	Tavangari, 2016 [18]	Ng et al., 2018 [11]	Fukami et al., 2018 [27]	Skoglar et al., 2018 [25]	Strajina et al., 2019 [26]
**Years included**	2007–2011	2008–2012	2012–2014	2008–2015	2006–2011	2006–2016
**Nr of patients with SBO-VA**	49	103	71	44	63	60
**Abdominal-wall Hernia**	Excluded	4/40 (10%)	Excluded	-	-	excluded
**Operative treatment**	34	40	43	7	63	50
**Adhesions**	37/49 (75.5%)	14/40 (35%)*	44/71 (62%)	7/ 7 (100%)*	19/63 (30%)	13/50 (26%)*
	• Laparotomy proven *n* = 25/49 (51%) • Exclusion of other causes *n* = 12	-	• Surgically proven *n* = 23 /43 (53%) • Exclusion of other causes *n* = 21		-	-
**Adhesion type** **Band/Matt**	-	-	Band, *n* = 19 Matt, *n* = 4	-	Band, *n* = 13 Matt, *n* = 7	Band, *n* = 4
**Malignancy**	*n* = 5 (10%)	*n* = 4/40 (10%)	*n* = 3 (4%)	-	*n* = 26 (41%) *Included CRC which was the most common	*n* = 8 (13%)
**Negative Laparotomy**	*n* = 3 (6.1%)	-	-	-	-	20/50 (40%)
Other causes*:	*n* = 4 (8%)	*n* = 10/40 (10%)	*n* = 24 (33%)	-	*n* = 8 (13%)	*n* = 11 (18%)
	Malignancy; *n* = 5 Meckel`s diverticulum; *n* = 1 Gallstone ileus; *n* = 1 NSIAD induced IBD; *n* = 1 Sclerosing encapsulating peritonitis; *n* = 1		Malignancy; *n* = 3 Meckel’s diverticulum; *n* = 1 Gallstone ileus; *n* = 5 Phytobezoar/foreign body; *n* = 3 Internal herniation; *n* = 4 Intussusception; *n* =4 Mesentery volvulus; *n* = 3 Stricture; *n* = 1	-	Malignancy; *n* = 26	Malignancy; *n* = 8
**CT performed**	58/62 (93.5%)	102/103 (99%)	69/72 (96%)	-	-	60/60 (100%)
**CT accuracy in compared to operative findings**	18/34 (52.9%)	-	32/42 (76%)	-	-	-

*Etiology only available for surgically treated patients

Most studies did not specify the type or the location of the adhesions. Skoglar et al. reported that 13 out of 20 patients (65%) with SBO-VA caused by adhesions had solitary band adhesion and 7 (35%) had more extensive matted adhesions [25]. In contrast, the study reported that the majority of patients with previous surgery had matted adhesions (67%) [25].

Other etiologies reported for SBO-VA include malignant or benign tumors, arising primarily from the small bowel (NETs, lymphoma, and carcinomas), mesentery and retroperitoneum, or a metastatic origin (most commonly from a colonic, ovarian, or prostatic origin). Based on the results from the operative findings in the included studies, malignancy as the cause of SBO-VA was encountered in 4–13% [6, 11, 26].

Other less common causes of SBA-VA include bezoar, small bowel volvulus, intussusception, Meckel’s diverticulum, gallstone ileus, internal hernia, and new-onset IBD [6, 18, 26]. Detailed results of the etiology of SBO-VA are found in Table 1.

Two studies reported negative laparotomies, where an apparent cause of SBO-VA could not be established in 6% and 40% of the study population respectively [6, 26].

### Diagnostics in SBO-VA

#### Initial assessment

As for SBO in general, one of the main priorities in the initial assessment of the patient with SBO-VA is to identify indications for emergent surgical exploration [8]. Indications for emergency operation are signs of peritonitis, strangulation, and ischemia. History taking and physical examination might also reveal clues about the etiology of the obstruction [28]. During physical examination, the physician should seek abdominal wall hernias, including groin hernias. The initial assessment should also comprise an evaluation of nutritional status and sequelae of SBO such as dehydration.

Laboratory tests should include blood count, CRP, lactate, electrolytes, BUN/creatinine, and coagulation profile; elevated CRP, leukocytosis with left shift, and elevated lactate might be helpful indicating the presence of peritonitis and bowel ischemia, although normal values cannot exclude ischemia [29, 30].

### Imaging studies

#### Abdominal plain radiography

There are no studies discussing the diagnostic value of plain radiography specifically in patients with SBO-VA. Abdominal plain radiography is still used as first-line imaging in a patient with suspected of SBO, but it has limited value as complementary to the initial clinical assessment [31]. Sensitivity and specificity of plain X-rays for SBO is approximately 60–70% [32, 33]. Moreover, plain radiographs do not provide additional information on the etiology of the obstruction nor the need to perform emergency surgery.

#### Water-soluble contrast

Water-soluble contrast agents (WSCA) imaging has an established place in the management of ASBO in cases where a CT scan is not required [8]. WSCA has both prognostic and potential therapeutic value in the management of patients with small bowel obstruction; several studies have shown promising results in the management of ASBO [34–36]. The appearance of contrast in the colon within 4–24 h after the administration had a sensitivity of 96% and specificity of 98% in predicting resolution of SBO with conservative therapy; if the contrast did reach the colon on an abdominal X-ray taken 24 h following administration of the contrast, this was highly indicative of a failure of non-operative management [34–36].

Only two studies reported on the use of WSCA imaging in SBO-VA [24, 27]. Collom et al. reported WSCA to have been used less frequently in patients with SBO-VA (35.6%), as compared to patients with SBO after previous surgery (52.2%) [24]. A potential explanation might be that an adhesive etiology in the case of SBO-VA cannot be assumed, requiring a CT to establish the cause of the obstruction. Nevertheless, in the study of Fukami et al., WSCA seems to be equally effective in 44 patients with SBO-VA compared to 794 patients with surgery in history [27]. There were no significant differences in the duration of nasogastric tube decompression between patients with and without virgin abdomen (2.2 versus 2.5 days, *p* = 0.40), and intervals until the initiation of oral intake were also comparable (2.4 versus 2.6 days, *p* = 0.55). The overall operative rate was 16% in the VA and 17% in the group with previous surgery (*p* = 1.000) [27].

#### Computed tomography

The ability of computed tomography (CT) to provide information related to the underlying cause of SBO, and predict the need for emergency surgery, makes CT the primary diagnostic tool of choice in patients with SBO [8, 32]. However, two of the included studies on SBO-VA reported that the accuracy of CT in establishing the exact cause of obstruction seems to be low (53% and 76%). A possible explanation for this finding is that cases of SBO-VA were included over many years and (parts of) these cohorts were not recent [6, 11]. The introduction of multidetector-row computed tomography (MDCT) has improved the diagnostic accuracy with high sensitivity and specificity for the etiology of SBO (87 and 90%, respectively) [37, 38].

CT scan plays a key role in the decision on the management of patients with SBO; its importance is not just in the confirmation of the diagnosis of SBO, but also in identifying the underlying cause of obstruction and predicting the need for emergency surgery. Moreover, CT scan is also able to provide information about an alternative diagnosis if no signs of bowel obstruction are present [28].

Findings on CT scan help define the potential location of the obstruction (e.g., high in the jejunum or deep in the pelvis), the grade of the obstruction, partial or complete, and may also identify a possible transition zone [39]. The use of water-soluble contrast optimizes the diagnostic value of CT scan, and X-ray can evaluate the progress of the contrast at 24 h after CT [8]. Findings on the CT scan that predict the need for operative management, include a closed-loop obstruction, markers of bowel ischemia (mesenteric edema, free intraperitoneal fluid), and the “small bowel feces sign”, and additional radiological scores can be used to predict the need for surgery [37, 40].

There is increasing evidence that adhesions are a major cause of SBO-VA, similar to SBO in general. Although adhesions are not directly visible even on CT, the absence of other etiologies and a transition zone are highly predictive for adhesions as the cause of the obstruction [8, 36, 41]. When adhesive etiology is established by CT, patients with SBO-VA can be treated according to the same management algorithms as other patients with ASBO.

### Management of SBO-VA

#### Initial decision making

Most studies report a high rate for surgery SBO-VA ranging from 39 to 83% [6, 11, 18, 24, 26]. This compares relative high to SBO in general, in which 70% is treated non-operatively and the indication of most operations is a failure of non-operative trial [8]. Only in the cohort of Fukami et al. a low rate of 16% operative treatment was reported with the use of WSCA [27]. Most SBO-VA cohorts did not precisely report on the indications for surgical exploration nor if surgery was chosen as the initial treatment. Possibly, the presumption that SBO-VA has different causes than adhesions that do not resolve with conservative management explains the relatively high rate of surgical treatment.

#### Operative management

Operative intervention can be on an urgent basis for patients with signs of bowel compromise or later intervention when non-operative management fails. This distinction in the indication for surgery was reported in only one of the studies on SBO-VA [11]. In 32 of 43 operated patients (74%) with SBO-VA in this cohort, surgical exploration was performed as the primary treatment [11]. It was not reported if there were signs of peritonitis or ischemia in these patients as the indication of surgery, nor was data related to bowel resections reported. In the remaining 11 patients in whom a conservative trial was started, 5 were operated on after failure of non-operative management [11].

The cohort of Collom et al. and Strajina et al. excluded patients with signs of compromised bowel [24, 26]. Nevertheless, the operative rates remained high, reporting 39% and 83% respectively to have been treated surgically [24, 26].

Fukami et al. reported a lower operative rate of 16% in patients with SBO-VA by applying WSCA according to the same protocol as in patients with SBO and previous surgery in history [27].

#### Surgical technique

Laparotomy was the surgical approach of choice in patients with SBO-VA [6, 11, 26].

Two studies reported results related to the laparoscopic approach. Ng et al. reported a laparoscopic approach to have been used in 5/43 patients. Three patients were converted to laparotomy, two for open adhesiolysis, and one for better assessment of small bowel viability [11]. Strajina et al. reported that surgical exploration was completed laparoscopically in 18 out of 50 operated patients with SBO-VA (35%); it was not reported if there were conversions from an initial laparoscopic approach [26]. A higher rate of negative explorations was reported following laparoscopy compared to open surgery [26].

The use of adhesion barriers can reduce recurrence rates in case of a small bowel obstruction caused by adhesions [8, 42]. None of the studies on SBO-VA reported on the use of barriers or other means to reduce the recurrence of adhesions.

#### Results of surgical treatment

A few studies reported that the cause of obstruction could not be identified even in some cases even with operative exploration [6, 11, 18, 25]. Strikingly Strajina et al. reported negative finding in as many as 20 (40%) of surgical explorations [26]. In only three of these patients, the etiology of obstructive symptoms was established during follow-up, including one case of a neuroendocrine tumor, a motility disorder, and angioedema [26].

Few data were available on the morbidity and mortality related to the surgical treatment of SBO-VA. Fukami et al. reported a zero mortality rate [27]. Strajina et al. reported 30-day morbidity in patients with a therapeutic exploration of 39% (11 patients), compared to a significantly lower rate of 10% in patients with negative laparotomies [26].

Recurrence rates after initial operative management in patients with SBO-VA ranged between 1 and 10%; the findings were listed in two of the studies as shown in Table 2 [11, 26].

**Table 2:** Recurrences after initial operative management in SBO-VA

Study	Operative management (n)		Recurrence (n)	Findings (n)
Ng et al., 2018 [11]	43/72		1	SB volvulus secondary to adhesions.
Strajina et al., 2019 [26]	49/60	Therapeutic 29/49	2	Not specified.
		Negative 20/49	3	1 Small bowel - NET 1 Motility disorder 1 ACE angioedema.

#### Non-operative management

Non-operative management was used in the treatment of SBO-VA patients in 17–87% of patients (Table 3) [6, 11, 18, 24, 26, 27]. Most studies did not explicitly report criteria for non-operative or operative management. Beardsley et al. relied on the absence of previously known abdominal disease and the absence of pathological findings on the CT in the diagnosis of adhesions by exclusion. In this study, non-operative treatment was successful in all patients in whom a trial of non-operative management was started [6]. In the study of Ng et al., non-operative management failed in 5 (17%) [11]. Collom et al. reported that the success of conservative management depended on the use of WSCA; in the group of patients who received WSCA, a 17% failure rate was reported, as opposed to 50% in the group without WSCA [24]. In accordance with their findings, Fukami et al. reported only one patient treated by non-operative management including WSCA required surgery for the failure of non-operative management.

**Table 3:** Operative and non-operative management in SBO-VA, and failure non-operative management

Study	Total number of patients with SBO-VA	Non-operative management	Failure of non-operative management
**Ng et al., 2018** [11]	72	29 (40%)	5—failed
**Beardsley et al., 2014** [6]	49	15/49 (30%)	Zero
**Tavangari, 2016** [18]	103	63 (61%)	Zero
**Collom et al., 2018** [24]	101	62 (61%)	No WSCA—50% operation With WSCA—17% operation
**Fukami et al., 2018** [27]	44	38 (87%)	1
**Strajina et al., 2019** [26]	60	10 (17%)	-

As with ASBO in patients with previous surgery, the cornerstone of the non-operative management is fasting, fluid and electrolytes replacement, and bowel decompression by nasogastric tube [27]. Detailed information such as the duration of the non-operative trial and type of decompression tubes was not reported. Two of the studies reported the use of WSCA in patients assigned for non-operative management [24, 27]. The protocol for administration of WSCA in the study of Collom et al. was not detailed [24]. In the cohort by Fukami et al., all patients who were assigned for non-operative management received 100 ml Gastrografin within 24 h after admission; abdominal X-rays were taken after WSCA administration, and the obstruction grade was classified into two major categories, complete obstruction group and incomplete obstruction [27].

#### Follow-up and recurrence

Recurrence during follow-up was not consistently reported. A few studies did mention recurrences though. In the study of Tavangari et al., 5 out of 63 non-operatively treated patients had a recurrence, all were treated successfully without intervention in the recurrence episode, and data related to further investigations and findings in the follow-up after those patients were not reported [18].

None of the studies reported the use of a pre-established follow-up and surveillance program for patients treated non-operatively.

Strajina et al. reported a median follow-up of 53 months after patients treated non-operatively; one patient had transverse colon cancer diagnosed at colonoscopy 3 months later; 2 patients suffered recurrent SBO, one patient required bowel resection due to endometriosis, and the other had resection of benign small bowel stricture [26].

Ng et al. reported that only 5/29 patients in the non-operative group had a colonoscopy or small bowel study in the follow-up, and the remaining 24 patients did not have any further evaluation. Interestingly, only two out of the 29 patients required readmission for recent SBO at follow-up. [11].

## Discussion

SBO-VA is a relatively uncommon subgroup of small bowel obstructions, and there are only a few reports dedicated to patients in this subgroup.

The available evidence however contradicts the opinion suggesting that most cases of SBO-VA have non-adhesion cause, and all patients with SBO-VA, therefore, require surgical exploration.

The data from the reviewed studies shows that etiology and treatment results for patients with SBO-VA are largely comparable to the results in patients with SBO after previous abdominal surgery. CT scan has a pivotal role in the assessment of SBO-VA to assess the etiology and to evaluate if the bowel is compromised, demanding early surgery. Moreover, modern high resolution CT is also useful in minimizing the risk of failure to detect a malignant cause. As with ASBO in general, the majority of cases with SBO-VA can be treated by non-operative trial initially. Nevertheless, a laparotomy remains indicated in case of a non-resolving obstruction.

Based on the findings in our review, SBO-VA mostly has a benign cause; this is in contrast to older literature and surgical textbooks that suggest malignancy as the main cause of obstruction in patients with a virgin abdomen [10, 43]. Possibly, improvements in imaging and diagnostics techniques in past decades have contributed to the diagnosis of abdominal pathologies, including malignancies, at an earlier stage, resulting in fewer cases presenting as a bowel obstruction. For example, recent studies have shown that improved diagnostics and screening programs have resulted in a stage shift in the diagnosis of colon carcinoma, with more tumors diagnosed at an early stage in both the USA and Europe [44, 45]. Although such epidemiological data is not available for small bowel disease, a similar trend might also be applicable.

It is clear from the presented data that an initial operative management was favored in SBO-VA; such an approach could expose patients to a high rate of avoidable laparotomies and associated morbidity. The data also suggested that the use of non-operative trial achieved a spontaneous resolution of the SBO in most of the patients; moreover, as Collom et al. showed, the use of WSCA significantly reduced the need for operative intervention. Following successful conservative treatment of SBO, diagnosis and management of potential underlying causes can be performed electively as needed.

Based on the etiologic findings in the presented studies, fear of the previously assumed high rate of malignancies is not justified. As a consequence, the need to perform surgical exploration in every patient with SBO-VA can also be waived. Nevertheless, non-invasive diagnostics with high accuracy for detection of malignancy or follow-up is mandatory as approximately one in ten cases of SBO-VA is still caused by malignancy.

## Conclusion

Although several aspects in diagnosis and treatment of SBO-VA have not been clearly described in the few available studies, the overall results are comparable to the results of studies on SBO in patients with previous abdominal surgeries in history. We therefore propose that patients with a virgin abdomen could be treated according to existing guidelines for SBO and ASBO [8].

## Data Availability

All data generated or analyzed during this study are included in this published article [and its supplementary information files].

## Abbreviations

SBO: Small bowel obstruction
SBO-VA: Small bowel obstruction in the virgin abdomen
ASBO: Adhesive small bowel obstruction
WSCA: Water-soluble contrast agents
CT: Computer tomography
MDCT: Multidetector-row computed tomography
